# Contextual adaptation of digital wellbeing interventions for young people: insights from a project in Saudi Arabia

**DOI:** 10.3389/fpsyt.2024.1455962

**Published:** 2025-01-31

**Authors:** Dahlia Aljuboori, Laura K. Clary, Saud Abdulaziz Alomairah, Michelle Colder Carras, Nazmus Saquib, Juliann Saquib, Fahad Albeyahi, Antonius J. Van Rooij, Anouk Tuijnman, Vincent G. Van der Rijst, Michelle R. Kaufman, Johannes Thrul

**Affiliations:** ^1^ Department of Mental Health, Johns Hopkins Bloomberg School of Public Health, Baltimore, MD, United States; ^2^ Department of Biomedical Sciences, University of Copenhagen, Copenhagen, Denmark; ^3^ Department of International Health, Johns Hopkins Bloomberg School of Public Health, Baltimore, MD, United States; ^4^ Sulaiman Alrajhi University, Al Bukairiyah, Saudi Arabia; ^5^ Global Digital Wellbeing program (Sync), King Abdulaziz Center for World Culture (Ithra), Dhahran, Saudi Arabia; ^6^ Department of Digital Media & Gambling, Trimbos Institute, Utrecht, Netherlands; ^7^ Department of Health, Behavior and Society, Johns Hopkins Bloomberg School of Public Health, Baltimore, MD, United States; ^8^ Sidney Kimmel Comprehensive Cancer Center at Johns Hopkins, Baltimore, MD, United States; ^9^ Centre for Alcohol Policy Research, La Trobe University, Melbourne, VIC, Australia

**Keywords:** digital wellbeing, digital media use, adolescence, intervention development, school-based intervention, culturally responsive interventions

## Abstract

In today’s world, the internet is seamlessly woven into every facet of our existence. This constant engagement with digital media has generated concerns about the negative effects of digital media use, especially among adolescents. These concerns have led to the development and testing of numerous digital wellbeing interventions that focus on adolescents’ digital media use. However, these interventions are lacking in the Middle East and North Africa, and specifically in Saudi Arabia, where digital media use is highly prevalent and frequent. Our research team is conducting a series of studies - literature reviews, stakeholder engagement work, and a nationwide survey of adolescent digital media use - to inform, develop, and ultimately test school-based digital wellbeing intervention for high school students in Saudi Arabia. The goal of this manuscript is to explain our process of informing and creating an intervention that builds on previously established, evidence-based approaches, and is also tailored to a particular context (e.g., Saudi Arabia). Moreover, we distill the lessons learned from each study and provide recommendations to assist others in developing tailored digital wellbeing interventions for contexts that have not been the focus of previous intervention development.

## 1 Introduction

We live in a world where digital media use – gaming, streaming, social media, online shopping, and chatting with friends – is increasingly a part of people’s daily life, regardless of country, culture, or socioeconomic status. In recent years, the COVID-19 pandemic spurred further digital media use, as people sought virtual connections as alternative avenues for in-person social interaction (1).

The proliferation and integration of digital media into many aspects of adolescents’ lives has led to both benefits and challenges. Benefits include increasing social interactions, helping individuals build community online, and providing access to information and education (2–4). Increases in digital media use, however, have coincided with decreases in beneficial health behaviors (e.g., physical activity), as well as a rise in the prevalence of negative mental health (e.g., depression, anxiety) and addictive behaviors, such as gaming, gambling, and problematic social media use (1). These benefits and risks demonstrate that careful balance of digital media use is needed to maximize digital wellbeing, the subjective experience of achieving a healthy balance in digital media and technology use, while avoiding problems from excessive usage or negative content (5). Thus, interventions to address digital wellbeing should be designed to maximize the benefits and minimize harms of using digital media and promote balance between online versus offline activities.

Most digital wellbeing interventions are developed and implemented in North America, Europe, and Asia with limited information on generalizability and adaptability to other parts of the world. Yet, adolescents in Africa and the Middle East experience the same or even higher rates of problematic digital media use. For instance, the prevalence of social media addiction is higher for adolescents in Africa, Asia, and the Middle East compared to adolescents in North America and Western and Northern Europe (6). Also, recent studies have shown an increased frequency of internet gaming disorder specifically among youth in Asian countries such as India, Nepal, and South Korea, and countries in the Middle East and North Africa (MENA) region, such as Egypt, Ethiopia, Morocco, Saudi Arabia, and Tunisia (7–12) Altogether, these studies show that there is an urgent need for effective digital wellbeing interventions around the world, particularly for those, like Saudi Arabia, who are experiencing a recent surge in digital media use.

When planning to implement interventions for a new cultural context, using an existing evidence-based intervention or intervention components can be more efficient than developing a new intervention. Intervention effectiveness, however, often depends on the specific cultural context, such as geographical, organizational, cultural, economic, ethical, legal, political considerations, and local practices (13). For instance, interventions implemented in collectivist cultures (i.e., societies that prioritize the needs of the community or family) should consider using strategies that integrate parent and community education and accountability.

Taken together, digital wellbeing interventions that are culturally specific are needed to improve outcomes in many countries around the world that are experiencing an unprecedented growth in digital media use. This paper presents lessons learned from adapting interventions for a specific cultural context using a project on improving digital wellbeing among young people in Saudi Arabia as a guiding example.

## 2 Project overview: improving digital wellbeing in Saudi Arabia

This manuscript is part of a larger project to improve digital wellbeing for adolescents in Saudi Arabia, an international collaborative effort consisting of researchers from the Johns Hopkins Bloomberg School of Public Health (United States), Sulaiman Al Rajhi University (Saudi Arabia), the Global Digital Wellbeing program (Sync) at King Abdulaziz Center for World Culture (Ithra; Saudi Arabia), and the Trimbos Institute (Netherlands). Through comprehensive formative research, with several sequential studies, we aim to create a culturally tailored intervention to promote digital wellbeing among young people in Saudi Arabia, culminating in a rigorous randomized controlled trial to test intervention efficacy. Below, we introduce the unique context of Saudia Arabia, as well as each of the studies and their purpose (**Table 1**), and initial lessons learned (**Table 2**). Publications with detailed findings on these studies are forthcoming.

**Table 1:** Project overview.

Project steps	Details
1. Literature review:	
Rapid review of prevention interventions	We conducted a rapid review to identify existing prevention strategies related to problematic digital media use, including internet addiction, smartphone addiction, and gaming disorders.
Reviews of problematic digital media use interventions	We are in the process of conducting two reviews to examine the efficacy of intervention approaches that aim to treat problematic digital media use, including a systematic review of primary studies and an umbrella review of existing systematic reviews.
2. Stakeholder engagement:	
Stakeholder survey	We collected survey data to engage a diverse group of stakeholders from Saudi Arabia (N=92), including young people, parents, policy makers, industry leaders, clinicians, educators, and digital media users and non-users. The survey aimed to gather their perspectives on digital media use and its impact within the Saudi context.
Focus groups and individual interviews	We conducted focus groups and individual stakeholder interviews (total N=58), to collect additional rich qualitative data on stakeholder perspectives and opinions on problems with digital wellbeing in Saudi Arabia and what type of interventions may work for the Saudi context.
3. Epidemiological survey:	
	We recently completed a comprehensive high school-based survey in seven major Saudi regions to assess the prevalence of digital media use and related life domains among students. This survey also examined the physical and mental health of high school students. These data will further inform intervention priorities and can be used for educational materials to inform young people and the general public.
4. Intervention development:	
Intervention mapping workshop	A multi-step intervention mapping workshop was held in the Netherlands, involving experts from Saudi Arabia, the Netherlands, and the US. The objective was to define the problem, identify desired changes, and establish feasible intervention goals.
Follow-up session in Saudi Arabia	The initial goals and objectives were refined in additional intervention mapping sessions with Saudi youth, educators, and local experts to further ensure the intervention will be culturally relevant and contextually appropriate.
Intervention development and adaptation	Intervention development and adaptation is currently in progress. Intervention components are based on digital balance intervention materials originally developed by researchers at the Trimbos Institute for delivery in the Netherlands. Further testing of intervention components will be done in focus groups.
5. Intervention test:	
	Moving forward, we plan to conduct a rigorous randomized controlled trial of our new intervention and will evaluate its feasibility and efficacy in improving digital wellbeing among young people in Saudi Arabia.

**Table 2:** Lessons learned and recommendations for developing or adapting digital wellbeing interventions to specific cultural contexts.

Strategy	Detailed approaches
Review the existing literature	• Ensure relevance of literature reviews by emphasizing transferability of results in research questions. • Use tested interventions from various contexts as a foundation. • Identify effective strategies that can be adapted.
Explore the specific context for application	• Investigate the cultural, social, and environmental context where the intervention will be applied. For example, for the context of Saudi Arabia this means a focus on cultural nuances, gender roles, environmental conditions, and religious practices. • Tailor the intervention to the local context for increased relevance and acceptability among the target audience.
Involve community stakeholders	• Early engagement with the community, authorities, and high-level informants is crucial for developing culturally sensitive and contextually appropriate interventions. • Conduct focus groups, feedback meetings, and workshops to build these partnerships, which are essential for long-term sustainability and feasibility.
Implement iterative development with feedback loops	• Use an iterative approach. • Incorporate continuous feedback from diverse stakeholders. • Include the target audience, educators, and policymakers. This dynamic process refines the intervention based on real-world experiences, enhancing its efficacy and scalability.

## 3 The Saudi Arabian context

Saudi Arabia has a population of over 36 million and occupies most of the Arabian Peninsula (14). The country is characterized by its harsh environmental conditions and vast deserts, including the Rub’ al Khali (Empty Quarter), the largest continuous sand desert in the world (15). Saudi Arabia is considered a high-income country and the largest free-market economy in the MENA region. It is the birthplace of Islam and home to a predominantly Muslim population, with Islamic heritage and Arabic collectivist customs defining its traditions and culture. Cultural values center collectivism, with loyalty and long-term commitment to family, extended family, or the larger community. Traditionally, this culture also used to be highly gender segregated in public spaces, and as such women tended to have limited public roles.

Recently, however, under the leadership of Crown Prince Mohammed bin Salman, Saudi Arabia has undergone unprecedented social reforms and technological innovations to enhance the nation’s global standing, diversify its economy, and improve its citizens’ quality of life (16, 17). This includes quality of life improvements for women, such as the ability to drive and to participate more fully in the workplace and community at large. Together, these efforts, alongside significant investments in the video game industry, the launch of the Esports World Cup, and advancements in digital transformation and the digital economy, mark a turning point in Saudi culture regarding technology use. Consequently, there has been a rapid increase in digital media usage across genders, ages, and income brackets, particularly among young people (18). Any intervention development process must address these societal transformations to ensure that interventions are relevant and effective for today’s youth in Saudi Arabia.

## 4 Studies and lessons learned

### 4.1 Literature review

We first conducted a rapid review of the literature to identify recent interventions that were tested to prevent problematic digital media use (19). As part of this literature review, we identified interventions aimed toward promoting healthy use of digital media or preventing problematic use that targeted children, adolescents, and young adults to age 25, and were either universal (targeting general community populations) and selective (targeting specific subgroups who have higher risk factors and/or symptoms, but no disorder). These preventative interventions were conducted in various settings, including schools, outpatient clinics, and on online platforms. The most common intervention components were education or training, which includes information about self-regulation, digital wellbeing education, media literacy/effects, mental health symptoms and coping (psychoeducation), or a combination of multiple approaches. The primary goals of these interventions were to prevent problematic use, promote digital wellbeing, and improve symptoms of internet gaming disorder (IGD) and problematic internet use (PIU). Included studies were conducted in a diverse range of countries, including Spain, the USA, Germany, and South Korea. However, there was an evident gap in research focused on many regions of the world, including the MENA region, the Gulf Cooperation Council (GCC) countries, and Saudi Arabia specifically. This underscores the critical need for localized knowledge and understanding to develop effective digital wellbeing interventions that are culturally relevant and contextually appropriate for young people in Saudi Arabia.

Based on our work on this review, we identified several critical factors for effective implementation that must be considered to ensure scalability and adaptability in different contexts, for example:

- **Intervention costs**: Interventions requiring trained professionals and clinicians can be more costly than online interventions, highlighting the need to consider resource availability and the potential efficacy of online interventions to address digital wellbeing.
- **Training and competence of those delivering the intervention**: Interventions that are delivered by individuals with minimal training can be more easily scalable, but face challenges due to the difficulties of providing individualized or culturally validated training.
- **Intervention protocols**: Protocols or intervention manuals are crucial for understanding existing interventions and their implementation but were not shared by many studies. This complicates the adaptation of interventions or intervention components to different contexts.

### 4.2 Stakeholder engagement

As part of our stakeholder engagement work, we collected *survey data* to engage a diverse group of stakeholders from Saudi Arabia (N=92), including young people, parents, policy makers, industry leaders, clinicians, educators, and digital media users and non-users. Moreover, we conducted *focus groups and individual stakeholder interviews* (total N=58), to collect additional qualitative data. Altogether, these interviews and focus groups gathered stakeholder perspectives on digital media use and its impact, including problems with digital wellbeing in Saudi Arabia and what type of interventions may work for the Saudi context.

Survey respondents reported high digital media use, with varying impacts on their wellbeing. Stakeholders generally agreed on the benefits of digital media in education and communication but expressed concerns about the negative impact of digital media on sleep, physical activity, and concentration. Stakeholders perceived children and adolescents as the primary target population for interventions and suggested that school programs for students and education programs for parents, potentially building on digital tools such as apps, would be the most effective interventions. Additionally, the stakeholder survey revealed that Saudi citizens have a high level of trust in the government, in contrast to some Western countries, such as the USA (20). Some participants believed that interventions could be highly effective when implemented through existing government platforms (e.g., Absher and Sehhaty). Absher is a widely used government service app that facilitates various administrative tasks, such as national identity and passport issuance, and Sehhaty is a health services app providing access to medical consultations and records. Focus group and individual qualitative interviews with stakeholders resulted in similar insights. Overall, the stakeholder engagement results pointed to the importance of intervention content targeted at young people and their broader families. We learned that engaging a variety of stakeholders is crucial for developing a culturally and contextually appropriate intervention for Saudi Arabia.

### 4.3 Epidemiological survey

We recently completed data collection in seven major Saudi regions to assess the prevalence of digital media use and related life domains among high school students (target N=3,000). The questionnaire assessed domains critical to understanding adolescent digital wellbeing, including: (1) digital media use (e.g., screen time, social media use, streaming services, video games, etc.); (2) perceived impact of digital media use on wellbeing, including physical (e.g., activity, sedentary behavior, sleep), mental (e.g., anxiety, depression), and social wellbeing (e.g., loneliness); and (3) strategies to regulate digital media use. We also collected data on participant demographics, self-reported academic performance, religiosity, and family digital media use, rules, and overall family climate. The questionnaires used in the survey include both validated measures (e.g., Pittsburgh Sleep Quality Index, FACES IV, WHOQOL-BREF, PHQ-4), and investigator-developed questions. Data analysis is ongoing, and results will further inform intervention priorities and can be used for educational materials to inform young people and the general public.

### 4.4 Intervention development

We conducted multiple steps of iterative intervention development based on the Intervention Mapping approach (21, 22). This included a 3-day in-person intervention mapping workshop in the Netherlands, involving experts from Saudi Arabia, the Netherlands, and the USA (23). The objective was to (1) understand the health problem, the population at risk, behavioral and environmental causes, and their determinants (logic model of the problem); (2) create objectives for changing determinants and specify intervention targets (logic model of the change); and (3) identify theory- and evidence-based behavior change methods and translate them into practical applications (program design). Following the initial workshop, we conducted additional in-person intervention mapping sessions in Saudi Arabia with Saudi youth, educators, and local experts to further ensure the intervention will be culturally relevant and contextually appropriate (24).

A key finding from this process was the significance of family dynamics in Saudi Arabia. The family-centric culture, characterized by strong familial ties and collective decision-making, necessitates the integration of the family unit in interventions. Engaging parents and even grandparents alongside young people is essential, contrasting with many Western contexts that often focus more on individual behavior change (25, 26). Parents also play a critical role in managing children’s digital media use habits. Effective interventions should, therefore, include components that educate and empower parents, such as workshops and community forums, or online content delivery. We recommend that these parental and family outreach components be organized through the school systems, so they can be holistically integrated with school-based interventions.

Additionally, public schools in Saudi Arabia were frequently mentioned as the ideal setting for implementing digital wellbeing programs, due to schools’ growing awareness of digital wellbeing issues. For instance, digital wellbeing topics, alongside subjects like digital citizenship, have recently been introduced into the school curriculum, emphasizing the importance of addressing these issues within the educational system. Although this is a crucial first step, we found no existing interventions in Saudi schools that aim to change digital media use behavior. The few existing behavior change interventions primarily focus on physical activity and nutrition, and not digital media use (27), but still can provide valuable insights for developing our digital wellbeing program. For instance, these existing interventions tended to combine multiple approaches including physical education, sports activities, counseling, and nutrition awareness, and engaged not just students, but also parents to reinforce health behavior change (27).

The lack of digital media use interventions implemented in this region confirmed that our intervention development and adaptation process has to rely on digital wellbeing intervention content that was developed and delivered in other regions of the world and needs to undergo important cultural adaptation to address the unique context of Saudi Arabia.

Other unique aspects of the Saudi Arabian cultural context also need to be carefully considered. As previously mentioned, Saudi Arabia is currently undergoing rapid technological transformations. Indicating that both preventative and intervention content should be integrated into school-based approaches. Also, rapid changes indicate that interventions should be flexible, and content reviewed frequently, so that new content, formats, and platforms can be introduced to the programming as needed. Transformations in gender roles and the changes/increases in women’s participation in many aspects of society also should be considered when adapting interventions to this context. Moreover, the climate conditions of Saudi Arabia need to be taken into account as well when designing interventions, in particular the extreme heat. Due to high temperature levels year-round, youth will be inside for a great portion of the day, and recommendations around physical activity as part of digital wellbeing need to consider this factor. The climate conditions might lead to specific recommendations around the time of day that physical activity is recommended, as well as suggestions or programming that are tailored to the heat, such as a focus on indoor physical activities.

Building on these findings, we are currently in the process of developing, adapting, and further focus-group testing intervention components, some of which are based on digital balance intervention materials originally developed by researchers at the Trimbos Institute for delivery in the Netherlands (28).

### 4.5 Intervention test

Moving forward, we plan to conduct a rigorous randomized controlled trial of our new intervention and will evaluate its feasibility and efficacy in improving digital wellbeing among young people in Saudi Arabia.

## 5 Discussion

The widespread digital media use among young people, especially after the COVID-19 pandemic, has raised concerns for adolescent mental and physical health (1). This, in turn, has led to the development and implementation of digital wellbeing interventions; however, some parts of the world, such as the MENA region, lack such interventions. Developing a new intervention or adapting components from existing interventions is a dynamic process and involves multiple steps: understanding the existing needs and cultural dynamics influencing the target population, thorough planning of the intervention, pilot testing in multiple settings, and incorporating feedback to address potential challenges (13, 29). Although we may not have reached saturation of themes with a relatively small number of participants in different stakeholder groups, specific strengths of this project include collecting both quantitative and qualitative data from stakeholders, working with a team of collaborators that are familiar with the Saudi context, and developing a multi-component intervention that addresses specific contexts unique to the culture of the region (e.g., a significant parent component). In the process of developing an intervention specific to the needs of this population, we generated recommendations that other researchers can use to develop or adapt digital wellbeing interventions to specific cultural contexts (see **Table 2**). The discussion below highlights some key takeaways from this project.

The literature review underscored the lack of digital wellbeing interventions that were developed and tested in the MENA region, the GCC countries, and Saudi Arabia, highlighting the need for localized knowledge to ensure cultural relevance of interventions. The stakeholder engagement work pointed to the primary target group of children and adolescents and highlighted opportunities for integrating the broader family into school-based interventions: for example, by including digital wellbeing materials in school curricula and, at the same time, developing education programs for parents. The high level of trust in the government among Saudi citizens, combined with the widespread use of national governmental apps, offers a unique opportunity to implement digital wellbeing interventions through trusted and well-utilized platforms. A combination of school-based interventions targeting students paired with content on widely used apps for parents could obtain high acceptance and adherence and ensure scalability of intervention rollout.

During the Intervention Mapping, we systematically integrated theory and evidence to comprehensively address digital wellbeing, including identifying and addressing potential mechanisms of change, such as psychoeducation, self-monitoring, social support, behavioral substitution, and others. Additionally, we ensured that our proposed intervention is contextually appropriate by involving iterative needs assessment steps, setting objectives, and selecting intervention methods most suited to meet those needs. Insights from this process further underscored the significance of family dynamics in Saudi Arabia; the family-centric culture, characterized by strong familial ties and collective decision-making, necessitates the integration of the family unit in interventions. In the next phase, we will continue to utilize the Intervention Mapping approach (22) to implement and evaluate culturally relevant programming. Continued involvement of diverse stakeholders (e.g., young people, parents, educators, government officials) in an iterative intervention development process with built-in feedback loops is crucial for ensuring that the intervention will meet the needs of the target population and can be implemented with feasibility.

## 6 Conclusion

Saudi Arabia presents a unique context for digital wellbeing interventions, inhabiting specific cultural, social, and familial dynamics as well as demonstrating significant investments in the digital economy and rapid digitalization. This project is one of the first systematic efforts to develop a culturally tailored digital wellbeing intervention in Saudi Arabia. This research is critical to advance intervention adaptations to multiple contexts and can be applied to intervention development more broadly in this field. Development of effective interventions is essential to improve digital wellbeing and a healthy balance between online versus offline activities, while avoiding problems from excessive digital media and technology use.

## Data Availability

The original contributions presented in the study are included in the article/supplementary material. Inquiries can be directed to the corresponding author.

## References

[1] Marciano L Ostroumova M Schulz PJ Camerini AL. Digital media use and adolescents’ Mental health during the covid-19 pandemic: A systematic review and meta-analysis. Front Public Health. (2022) 9. doi: 10.3389/fpubh.2021.793868 PMC8848548 35186872

[2] Naslund JA Bondre A Torous J Aschbrenner KA. Social media and mental health: benefits, risks, and opportunities for research and practice. J Technol Behav Sci. (2020) 5:245–57. doi: 10.1007/s41347-020-00134-x PMC7785056 33415185

[3] Wilson C Stock J. ‘Social media comes with good and bad sides, doesn’t it?’ A balancing act of the benefits and risks of social media use by young adults with long-term conditions. Health (Lond). (2021) 25:515–34. doi: 10.1177/13634593211023130 PMC8424608 34080463

[4] Alsanea FA. Digital technology and its impact on the social change of the Saudi family. Netw Intell Stud. (2023) 21):41–9. Available online at: https://ideas.repec.org/a/cmj/networ/y2023i21p41-49.html.

[5] Vanden Abeele MMP. Digital wellbeing as a dynamic construct. Commun Theory. (2021) 31:932–55. doi: 10.1093/ct/qtaa024

[6] Cheng C Lau Yc Chan L Luk JW. Prevalence of social media addiction across 32 nations: Meta-analysis with subgroup analysis of classification schemes and cultural values. Addict Behav. (2021) 117:106845. doi: 10.1016/j.addbeh.2021.106845 33550200

[7] Khrad H Marhoomi A Alkhiri A Alshomrani A Bajabir D Mosli M. Prevalence of Internet Gaming Disorder among Saudi Arabian university students: relationship with psychological distress. Heliyon. (2022) 8. doi: 10.1016/j.heliyon.2022.e12334 PMC9798179 36590512

[8] Alsaad AJ Alabdulmuhsin FM Alamer ZM Alhammad ZA Al-Jamaan KA Al-sultan YK. Impact of the COVID-19 pandemic quarantine on gaming behavior among children and adolescents in the Eastern Province of Saudi Arabia. Int J Med Dev Ctries. (2021) 5:1007–7. doi: 10.24911/IJMDC.51-1610894250

[9] Shrestha MV Shrestha N Sharma SC Joshi SK. Gaming Disorder among Medical College Students during COVID-19 Pandemic Lockdown. Kathmandu Univ Med J KUMJ. (2020) 18:48–52. doi: 10.3126/kumj.v18i2.32956 33605238

[10] Balhara YPS Kattula D Singh S Chukkali S Bhargava R. Impact of lockdown following COVID-19 on the gaming behavior of college students. Indian J Public Health. (2020) 64:172. doi: 10.4103/ijph.IJPH_465_20 32496250

[11] Kim D Lee J. Addictive Internet Gaming Usage among Korean Adolescents before and after the Outbreak of the COVID-19 Pandemic: A Comparison of the Latent Profiles in 2018 and 2020. Int J Environ Res Public Health. (2021) 18:7275. doi: 10.3390/ijerph18147275 34299725 PMC8305932

[12] Endomba FT Demina A Meille V Ndoadoumgue AL Danwang C Petit B. Prevalence of internet addiction in Africa: A systematic review and meta-analysis. J Behav Addict. (2022) 11:739–53. doi: 10.1556/2006.2022.00052 PMC9872524 35984734

[13] Moore G Campbell M Copeland L Craig P Movsisyan A Hoddinott P. Adapting interventions to new contexts—the ADAPT guidance. BMJ. (2021) 374:n1679. doi: 10.1136/bmj.n1679 34344699 PMC8329746

[14] World Health Organization (WHO). Saudi Arabia. (2024). Available online at: https://data.who.int/countries/682 (Accessed June 25, 2024).

[15] Encyclopedia Britannica. Saudi Arabia | History, Map, Flag, Capital, Population, & Facts | Britannica (2024). Available online at: https://www.britannica.com/place/Saudi-Arabia (Accessed June 11, 2024).

[16] Saudi Vision 2030. (2024). Available online at: http://www.vision2030.gov.sa/en/ (Accessed June 26, 2024).

[17] Forster K Cena M. *Saudi crown prince seeks soft power in game hub Japan*. The Japan Times. (2024). Available at: https://www.Japantimes.co.jp/news/2024/05/18/Japan/saudi-Japan-mohammed-bin-salman/ (Accessed June 25, 2024).

[18] Saquib J. Internet addiction among Saudi Arabian youth. Int J Health Sci. (2020) 14:1–2. Available online at: https://pmc.ncbi.nlm.nih.gov/articles/PMC7069663/. PMC7069663 32206053

[19] Colder Carras M Aljuboori D Shi J Date M Karkoub F García Ortiz K. Prevention and health promotion interventions for young people in the context of digital well-being: rapid systematic review. J Med Internet Res. (2024) 26:e59968. 39693138 10.2196/59968 PMC11694046

[20] Almutairi AF BaniMustafa A Alessa YM Almutairi SB Almaleh Y. Public trust and compliance with the precautionary measures against COVID-19 employed by authorities in Saudi Arabia. Risk Manag Healthc Policy. (2020) 13:753–60. doi: 10.2147/RMHP.S257287 PMC7354916 32753988

[21] Eldredge LKB Markham CM Ruiter RAC Fernández ME Kok G Parcel GS. Planning Health Promotion Programs: An Intervention Mapping Approach. 4th edition. San Francisco, CA: Jossey-Bass (2016). 704 p.

[22] Fernandez ME Ruiter RAC Markham CM Kok G. Intervention mapping: theory- and evidence-based health promotion program planning: perspective and examples. Front Public Health. (2019) 7:209. doi: 10.3389/fpubh.2019.00209 31475126 PMC6702459

[23] Alomairah S Tuijnman A Thrul J. Intervention Mapping Workshop ‘Digital Balance Intervention Saudi Arabia. OSF (2024). Available online at: https://osf.io/n7ftk/.

[24] Alomairah S Tuijnman A Thrul J. Intervention Mapping sessions (Sync Summit 2024) Digital Wellbeing Intervention for Saudi Arabia. OSF (2024). Available online at: https://osf.io/jx9u5/.

[25] Al-Khraif R Abdul Salam A Abdul Rashid MF. Family demographic transition in Saudi Arabia: emerging issues and concerns. SAGE Open. (2020) 10:2158244020914556. doi: 10.1177/2158244020914556

[26] Almudhee SN Al Saigul AM Sulaiman A. Parenting style frequency and their sociodemographic determinants in Buraidah City, Qassim, Saudi Arabia. Cureus. (2023) 15:e41388. doi: 10.7759/cureus.41388 37546027 PMC10401485

[27] Elfaki FAE Khalafalla HEE Gaffar AM Moukhyer ME Bani IA Mahfouz MS. Effect of healthy lifestyle interventions in schools of Jazan City, Kingdom of Saudi Arabia: a quasi-experimental study. Arab J Nutr Exerc AJNE. (2020), 5:1–14. doi: 10.18502/ajne.v5i1.6911

[28] Trimbos-instituut. Digitale balans (2024). Available online at: https://www.trimbos.nl/kennis/gamen-gokken/mediawijsheid-en-digitale-balans/ (Accessed June 26, 2024).

[29] O’Cathain A Croot L Duncan E Rousseau N Sworn K Turner KM. Guidance on how to develop complex interventions to improve health and healthcare. BMJ Open. (2019) 9:e029954. doi: 10.1136/bmjopen-2019-029954 PMC6701588 31420394

